# Tyrosine kinase signaling-independent MET-targeting with CAR-T cells

**DOI:** 10.1186/s12967-023-04521-9

**Published:** 2023-10-01

**Authors:** Anna Qin, Yuan Qin, Joseph Lee, Anna Musket, Mingyao Ying, Giedre Krenciute, Francesco M. Marincola, Zhi Q. Yao, Phillip R. Musich, Qian Xie

**Affiliations:** 1 Department of Biomedical Sciences, Quillen College of Medicine, East Tennessee State University, Johnson City, TN 37614 USA; 2 Department of Surgery, Quillen College of Medicine, East Tennessee State University, Johnson City, TN 37614 USA; 3 Department of Neurology, Hugo W. Moser Research Institute at Kennedy Krieger, Johns Hopkins University School of Medicine, Baltimore, MD USA; 4 Department of Bone Marrow Transplantation and Cellular Therapy, St. Jude Children’s Research Hospital, Memphis, TN 38105 USA; 5 Sonata Therapeutics, Watertown, MA 02472 USA; 6 Department of Internal Medicine, Quillen College of Medicine, East Tennessee State University, Johnson City, TN 37614 USA; 7 Center of Excellence for Inflammation, Infectious Disease and Immunity, Quillen College of Medicine, East Tennessee State University, Johnson City, TN 37614 USA

**Keywords:** Hepatocellular carcinoma, MET tyrosine kinase receptor, Programmed cell death protein 1 (PD-1), Chimeric antigen receptor T cell therapy, Cancer immunotherapy

## Abstract

**Background:**

Recent progress in cancer immunotherapy encourages the expansion of chimeric antigen receptor (CAR) T cell therapy in solid tumors including hepatocellular carcinoma (HCC). Overexpression of MET receptor tyrosine kinase is common in HCC; however, MET inhibitors are effective only when MET is in an active form, making patient stratification difficult. Specific MET-targeting CAR-T cells hold the promise of targeting HCC with MET overexpression regardless of signaling pathway activity.

**Methods:**

MET-specific CARs with CD28ζ or 4-1BBζ as co-stimulation domains were constructed. MET-CAR-T cells derived from healthy subjects (HS) and HCC patients were evaluated for their killing activity and cytokine release against HCC cells with various MET activations in vitro, and for their tumor growth inhibition in orthotopic xenograft models in vivo*.*

**Results:**

MET-CAR.CD28ζ and MET-CAR.4-1BBζ T cells derived from both HS and HCC patients specifically killed MET-positive HCC cells. When stimulated with MET-positive HCC cells in vitro, MET-CAR.CD28ζ T cells demonstrated a higher level of cytokine release and expression of programmed cell death protein 1 (PD-1) than MET-CAR.4-1BBζ T cells. When analyzed in vivo, MET-CAR.CD28ζ T cells more effectively inhibited HCC orthotopic tumor growth in mice when compared to MET-CAR.4-1BBζ T cells.

**Conclusion:**

We generated and characterized MET-specific CAR-T cells for targeting HCC with MET overexpression regardless of MET activation. Compared with MET-CAR.4-1BBζ, MET-CAR.CD28ζ T cells showed a higher anti-HCC potency but also a higher level of T cell exhaustion. While MET-CAR.CD28ζ is preferred for further development, overcoming the exhaustion of MET-CAR-T cells is necessary to improve their therapeutic efficacy in vivo.

**Supplementary Information:**

The online version contains supplementary material available at 10.1186/s12967-023-04521-9.

## Background

Hepatocellular carcinoma (HCC) is a leading cause of cancer mortality due to lacking effective therapeutics [1]. While hepatitis B or C virus (HBV/HCV) infection is a  major cause of HCC, aberrant activation of c-Met oncogene, the receptor of hepatocyte growth factor (HGF), plays an important role in HCC initiation and progression [2, 3]. Experimentally, overproduction of the HBV L envelope protein leads to the formation of HBV surface antigen particles that accumulate in high concentrations in hepatocytes and ultimately induce carcinogenesis [4]. This process is enhanced by HGF stimulation, leading to HCC with a more malignant phenotype [5]. Clinically, overexpression of MET receptor occurs in approximately 40–50% of HCC patients, and correlates with poor survival [6, 7]. As such, MET is an important target for treating advanced HCC [1, 8].

In patients with advanced HCC, conventional chemotherapy is overall futile. Targeted therapeutics are preferable due to their specificity. Sorafenib is the only FDA-approved multi-target tyrosine kinase inhibitor (TKI) for standard care of HCC, but has a limited survival benefit [9]. Since MET became a promising anti-cancer target, several small molecule inhibitors and antibodies were tested in clinical trials against advanced HCC [8, 10]. Recently, the FDA approved several additional oral TKIs, including cabozantinib, a non-selective ATP competitor targeting MET, VEGFR2, and AXL. However, the overall improvement of survival is limited to 2–5 months [11, 12]. Particularly, cabozantinib is FDA approved (as a second-line treatment) for patients with HCC previously treated with sorafenib, but long-term toxicity remains a concern [11–14]. Because MET downstream signaling overlaps with other receptor tyrosine kinases (RTK), emerging challenges for MET inhibitors include acquired resistance and tumor recurrence [15, 16]. Clinical trials indicate that effective MET inhibitors require ongoing MET activation in tumor growth [17]. Thus, biomarkers for selecting suitable patients are key to treatment success. Given recent breakthroughs in cancer immunotherapy, the use of genetically-modified T cells expressing chimeric antigen receptors (CAR-T) is becoming a practical approach for treating HCC [18]. MET-specific CAR-T cells function through T cell signaling rather than RTK activity, therefore may overcome MET-TKI-mediated tumor resistance, and will be independent of previous treatments.

We previously reported differential responses to MET-targeting TKIs and neutralizing antibodies using cancer cell lines bearing different levels of MET expression [19–21]. The anti-MET monoclonal antibody (MetMab) potently inhibited HGF-dependent tumor growth, but showed no effect on tumor cells harboring MET amplification (MET^*amp*^), despite significant overexpression of MET protein [21]. Phase III clinical trials of MetMab were discontinued due to lack of efficacy.

Here, we generated and characterized MetMab-based MET-specific CAR-T cells and tested their expansion, persistence and therapeutic efficacy for targeting HCC with MET aberrations both in vitro and in vivo. MET-CAR-T cells with different co-stimulatory signaling modules are compared for their efficacy against HCC. Moreover, MET-CAR-T cells derived from both healthy subjects (HS) and HCC patients were analyzed.

## Materials and methods

### Cell lines

Human HCC cells C3A and SNU398 are from the American Tissue Type Collection; JHH5 is from the Japanese Collection of Research Bioresources. MHCC97H is from Fudan University Liver Cancer Institute [20]. MHCC97H cells with stable mCherry expression (MHCC97H^*mCherry*^) were generated by transfection of pmCherry-N1 (Clonetech) plasmid using Lipofectamine 2000 (Invitrogen) followed by stable clonal selection. SNU398 cells were grown in RPMI-1640 with 10% FBS. MHCC97H and C3A were grown in DMEM with 10% FBS. JHH5 was grown in Williams E with 10% FBS. Human whole blood from healthy donors was purchased from Physician Plasma Alliance, Grey, TN. Patients diagnosed with HCC were enrolled at the Johnson City Medical Center for whole blood collection. This study was approved by East Tennessee State University (ETSU) College of Medicine IRB. Upon collection, peripheral blood mononuclear cells (PBMCs) were isolated by Ficoll (GE Healthcare Bio-Science AB, Uppsala, Sweden) density gradient centrifugation and stored in liquid nitrogen [22, 23].

### Generation of specific MET-targeting CAR-T cells

**Fig. 1:**
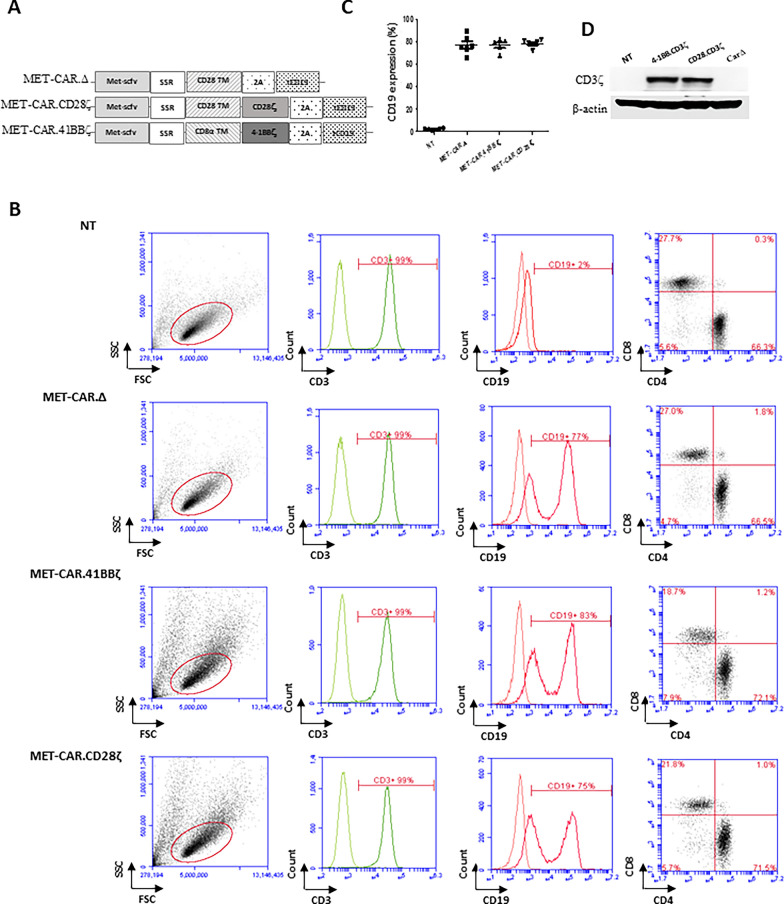
MET-CAR-T cell production and characterization. **A** MET-CAR structure. The specific MET-targeting scFv domain is linked with a TCR transmembrane (TM) domain (CD28 TM or CD8α TM) using a SSR. MET-CAR.CD28ζ and MET-CAR.4-1BBζ vectors use a CD3ζ domain (CD28ζ or 4-1BBζ, respectively) as the TCR signaling module. MET-CARΔ vector is a negative control that does not have the CD3ζ domain. All vectors have a 2A sequence linked to CD19 as an expression marker for measuring T cell transduction efficacy using flow cytometry. **B** CD3^+^, CD4^+^, CD8^+^ and CD19^+^ expression in NT and MET-CAR-T cells as determined by flow cytometry. **C** Overall MET-CAR-T cell transduction efficacy determined by percentage of cells with CD19 expression (%). **D** Total CD3ζ expression in MET-CAR-T cells measured by Western blotting

The retroviral-based CAR vectors contained a short non-signaling spacer region (SSR) to link CD28ζ or 4-1BBζ signaling modules (CAR.SSR.CD28ζ and CAR.SSR.4-1BBζ) and a control vector without a T cell receptor (TCR) endodomain (CAR.SSR.Δ) were provided by Dr. Stephen Gottschalk (St. Jude Children’s Research Hospital) [24]. The single chain variable domain coding sequence derived from MetMab [25] was synthesized and cloned into retroviral-based CAR vectors which encode CD19 as a marker for measuring T cell transduction efficacy (Fig. 1A). MET-CAR retroviral particles were produced via transient transfection of HEK 293 T cells. To produce MET-CAR-T cells, PBMCs isolated from HS or HCC patients were stimulated with anti-CD3/CD28 antibodies (Miltenyl Biotec) in the presence of IL-7 (10 ng/ml, Miltenyl Biotec) and IL-15 (5 ng/ml, Miltenyl Biotec) to expand CD3^+^ T cells for transduction on RetroNectin (Takara Bio)-coated plates [24]. To determine MET-CAR-T cell proliferation in vitro*,* five days after transduction,  MET-CAR-T cells were seeded in 24-well plates at 2.5 × 10^5^ cells/well in the presence of IL-7 (10 ng/ml) and IL-15 (5 ng/ml). Cells were counted using Countless II (Thermofisher) every other day with duplicates for each time point.

### Western blot

To test MET-CAR mediated T cell activation, MET-CAR-T cells were harvested 72 h after MET-CAR transduction and lysed in RIPA buffer (Thermo Fisher Scientific, Rockford, IL) containing protease and phosphatase inhibitors (Thermo Fisher Scientific, Rockford, IL). To test MET signaling activation in HCC cells, MHCC97H, JHH5, C3A, and SNU398 cells were cultured in 10-cm dishes until 80% confluence followed by cell lysis. Protein concentration was determined using the DC protein assay kit (Bio-Rad, Hercules, CA). 60 µg of total protein were run on a 10% SDS-PAGE gels (Life Technologies, Carlsbad, CA) and transferred to polyvinylidene difluoride membranes (Life Technologies, Carlsbad, CA) for antibody probing. Antibody-protein complexes were detected using SuperSignal West Dura Extended Duration Substrate (Thermo Fisher Scientific, Rockford, IL). Images were taken using ChemiDoc (Bio-Rad, Hercules, CA). We used HRP-labeled antibodies against human Met (clone 25H2), phospho-Met (Y1234/1235), AKT, phospho-AKT (S473), p42/44 MAPK, phospho-p42/44 MAPK (T202/Y204), GAPDH (14C10) (Cell Signaling Technology, Danvers, MA). Secondary antibodies used were goat anti-rabbit IgG-HRP and goat anti-mouse IgG-HRP (Santa Cruz Biotechnology).

### Flow cytometry analysis

To measure MET-CAR transduction efficacy, cells were collected on day 5 after transduction, washed with PBS containing 1% FBS, and incubated with CD3 and CD19 antibodies for 30 min followed by analysis using a flow cytometer (Acuri C6 + , BD). To determine the subsets of MET-CAR-T cells, CD19^+^ T cells were gated for  CD4^+^ and CD8^+^ populations. To determine programmed cell death protein 1 (PD-1) upregulation in MET-CAR-T cells, non-transduced (NT) or MET-CAR-T cells were co-cultured with MHCC97H cells in 24-well plates (10^5^ cells/well) at a 1:1 ratio for 3 days. At day 0 (before co-culture) and day 3, NT (gated by CD3^+^) and MET-CAR-T cells (gated by CD19^+^) were harvested to measure the PD-1 expression using flow cytometry analysis. Antibodies and isotype controls used in the analysis include: CD3 FITC (clone HIT3A, BD), CD19 PE (clone HIB19, BD), CD19 FITC (clone HIB19, BD), CD4 PE (clone RPA-T4, BD), CD8 APC (clone RPA-T8, BD), PD-1 Cy7(Clone EH12.1, BD), IgG2b κ FITC (clone 27-35, BD), IgG1 PE (clone MOPC-21, BD), IgG2b κ PE-Cy7 (clone 27-35, BD), and IgG1 APC (clone P3.6.2.8.1, Invitrogen, San Diego, CA).

### Confocal microscopic imaging

To visualize MET-CAR-T cell-mediated killing activity, MHCC97H^*mCherry*^ cells were seeded in 3-cm glass-bottom dishes at 5 × 10^5^ cells/well and grown at 37 °C for 24 h before co-culture with MET-CAR-T cells. Prior to co-culture, MET-CAR-T cells were stained with a mitochondrial fluorescence dye (Mitotraker Green FM, Thermofisher) at 300 nM for 30 min, washed with PBS, resuspended with culture medium, followed by co-culture with MHCC97H^*mCherry*^ cells at a 10:1 Effector T cell:Tumor cell (E:T) ratio for an additional 24 h. Three z-stack images were sequentially acquired every 10 min under mCherry (ex/em = 550–660), mitotracker Green (ex/em = 488/549) and differential interference contrast (DIC) channels under a confocal microscope (Letica TCS SP8).

### MET-CAR-T cell-mediated HCC cytotoxicity

MHCC97H, JHH5, C3A, or SNU398 cells were seeded in 96-well plates at 2 × 10^4^ cells/well and grown at 37 °C for 24 h. At day 2, NT and MET-CAR-T cells generated using PBMCs derived from the same HS or HCC patients were added into each well at 10:1, 5:1, 2.5:1, and 1.25:1 E:T ratios for co-culture with triplicates for each E:T ratio. After another 24 h, effector T cells were carefully washed out using PBS. The viability of tumor cells was determined using the MTS assay (Promega).

### Multi-panel cytokine release analysis

To prepare conditioned medium, MHCC97H cells were seeded in 24-well plates at 1 × 10^5^ cells/well and grown overnight followed by co-culture with NT and MET-CAR-T cells generated using PBMCs from the same HS (n = 4) or HCC patients (n = 7) at E:T = 2:1 ratio. After another 24 h, medium was collected and spun at 13,000 × rpm for 5 min (Sorvall RT7 Plus) and the supernatant fraction was stored at − 80 °C for human cytokine Multi-Analyte Profile A (MAP) analysis to determine the concentrations 16 cytokines released from each type of MET-CAR-T cells (Rules-Based Medicine, Austin, Texas). Python data visualization libraries Matplotlib 3.5.3 and Seaborn 0.12.1 were used to generate the heat-map of cytokine expression levels using the log2 fold change value, which is measured by comparing each cytokine expression level of MET-CAR-T cells to that of the paired NT cells. Student t test (p < 0.05) was used to compare the cytokine expression levels between HS and HCC groups. Paired t test (p < 0.05) was used to compare the cytokine expression levels between MET-CAR.CD28ζ and MET-CAR.4-1BBζ T cell groups.

### Therapeutic efficacy of MET-CAR-T cells against HCC orthotopic tumor growth in vivo

Athymic nude mice and NOD/SCID gamma (NOD.CB17-Prkd^scid^ IL2rg^tm1^/Bcgen) mice at about six weeks of age (Envigo) were used to evaluate the treatment for HCC orthotopic tumor growth using MET-CAR-T cells. Briefly, MHCC97H^luc+^ cells (5 × 10^5^) were used to initiate subcutaneous (SQ) tumor growth for liver implantation into the host mice to initiate intrahepatic tumor growth. 4–5 days after surgery, mice bearing tumors were randomized into experimental groups (n = 8) according to the bioluminescence imaging (BLI) intensity to receive a one-time treatment of MET-CAR-T cells (5 × 10^6^) through intravenous (i.v.) injection. Intrahepatic tumor growth was measured by BLI once a week. To perform BLI, each mouse received intraperitoneal injection of 100 µl luciferin (15 mg/ml, Biosynth). After 10–15 min, mice under anesthesia with isoflurane were imaged using an optiMAX imager (Precision Medicine). To evaluate the effectiveness of treatment, the average BLI signal intensity at each time point was analyzed with the Student’s t test (p < 0.05). All studies involving animals were performed at ETSU animal facility and are approved by the ETSU Institutional Animal Care and Use Committees.

### MET-CAR-T cell expansion and persistence in vivo

MHCC97H cells were SQ injected into NOD/SCID gamma mice to initiate tumor growth. When average tumor size reached 150 mm^3^, mice were randomized into 2 groups (MET-CAR.CD28ζ vs. MET-CARΔ, n = 5) to receive MET-CAR-T cells (5 × 10^6^) co-transduced with a GFP-Luc plasmid through intra-tumoral injection. CAR-T cells were monitored using BLI. Tumor size was measured by caliper every other day.

### Statistical analysis

GraphPad Prism 8 software (GraphPad software, La Jolla, CA) was used for statistical analysis. MET-CAR-T cell killing activity against HCC cells in vitro, PD-1 level before and after co-culture with MHCC97H cells, and BLI signal intensity at each time point in vivo were analyzed with Student’s *t* test (p < 0.05). Paired *t* test (p < 0.05) was used to compare the PD-1 upregulation in MET-CAR.4-1BBζ and MET-CAR.CD28ζ T cells co-cultured with MHCC97H cells.

## Results

### MET-CAR-T cell generation

We previously showed that HCC cells bearing different MET activation profiles respond to MET inhibitors differently [20, 21]. In particular, MetMab significantly blocked HGF-dependent HCC tumor growth, but failed to inhibit growth of tumors with HGF-independent MET activation and overexpression [21]. We, therefore, questioned whether expressing the MET binding domain of the MetMab antibody on the surface of T cells may generate MET-targeting CAR-T cells that are able to kill MET-overexpressing HCC cells regardless of MET pathway activation, since MET-CAR-T cells would rely on T cell functions which is independent of MET activation. To test this notion, two CAR vectors were constructed containing the scFv domain of MetMab along with costimulatory modules (MET-CAR.CD28ζ, MET-CAR.4-1BBζ). A CAR vector without costimulatory domain and CD3ζ signaling module (MET-CARΔ) served as a negative control (Fig. 1A). MET-CAR transduction efficiency (CD19%) ranged from 70 to 90% for all MET-CAR constructs (Fig. 1B, C). The transduction efficacy was also confirmed by western blot using CD3ζ as a marker, which was significantly up-regulated in both MET-CAR. CD28ζ- and MET-CAR.4-1BBζ-transduced T cells comparing with NT or MET-CARΔ-transduced T cells (Fig. 1D). Recent studies identified higher CD4^+^ T cell than CD8^+^ T cell subsets in final CAR-T cell products with differentiated target cell killing activities [26, 27]. Quantifying subtypes in NT (gated on CD3^+^ T cells) or MET-CAR-T cells (gated on CD19^+^ T cells), we also found similar T cell subset profiles, indicating that MET-CAR-T cell are a mixture of CD4^+^ and CD8^+^ T cells with CD4^+^ T cells constituting the major proportion, regardless of MET-CAR constructs (Fig. 1B right panels).

### MET-CAR-T cells specifically target HCC cells with MET overexpression regardless of MET activation

**Fig. 2:**
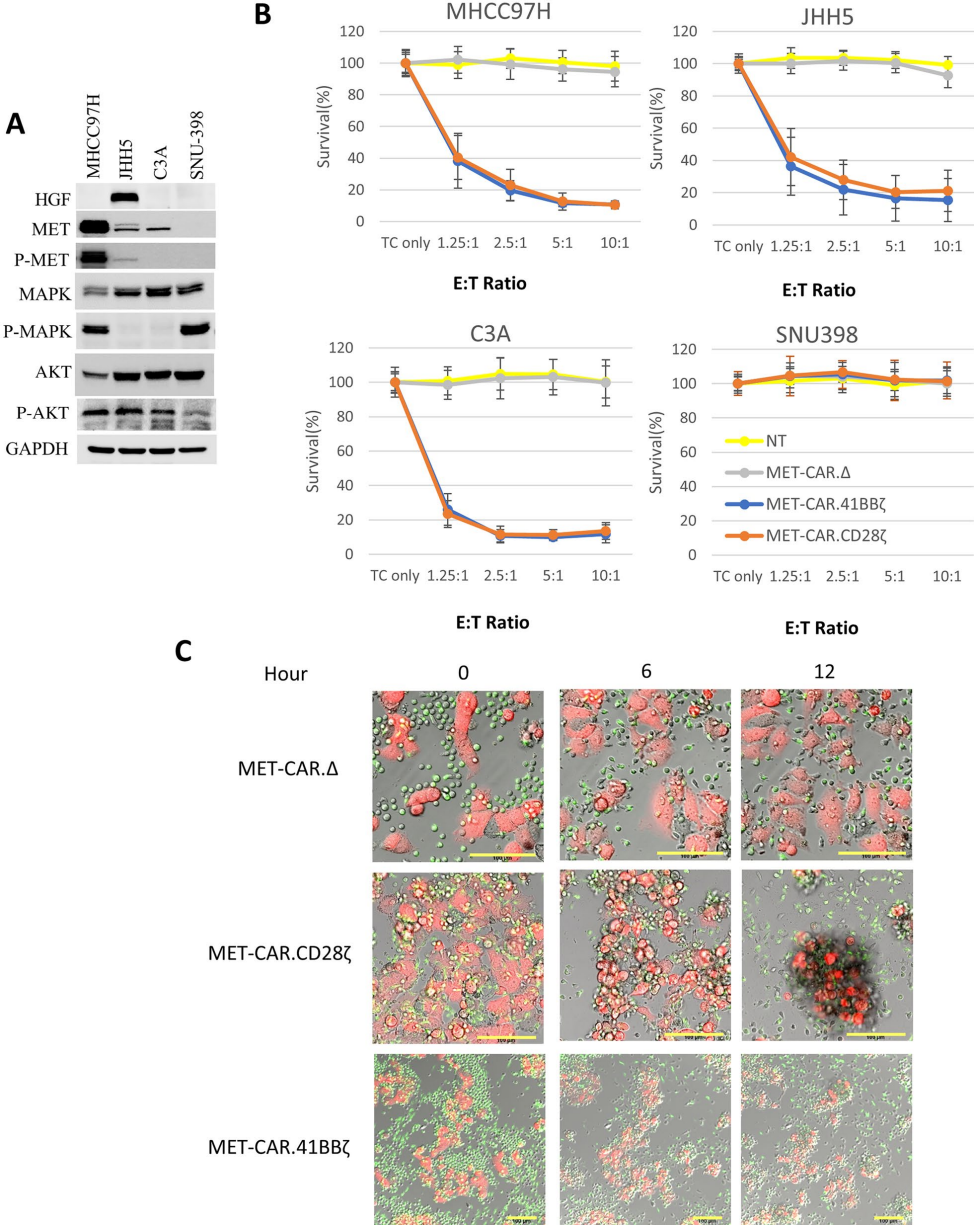
MET-CAR-T cells specifically kill HCC cells with MET overexpression in vitro. **A** HGF/MET expression level and pathway activity in MHCC97H, C3A, JHH5, and SNU398 (MET negative) cells. **B** MET-CAR-T^HS^ cell killing activity against MHCC97H, C3A, JHH5, and SNU398 cells in vitro. TC refers to tumor cells only. The MTS assay measures tumor cell viability. Survival (%) = (OD value of treated samples-OD value of blank)/ (OD value of TC-only samples-OD value of blank). Results were averaged from 5 independent experiment with PBMCs from healthy donors (n = 5). Triplicates were used for each experiment. Bracketed bar refers to standard deviation. **C** Confocal imaging of MHCC97H^*mCherry*^ and MET-CAR-T cell co-culture. MET-CAR-T cells (green) and MHCC97H^*mCherry*^ cells (red) were co-cultured at E:T = 10:1 ratio. After 24 h sequential z-stack images were acquired under mCherry (ex/em = 552/589–660), MitoTracker Green (ex/em = 488/497–549) and DIC. Scale bar refers to 100 μm

Four HCC cell lines with variable MET/MAPK/AKT pathway expression and activation levels were selected for testing specific MET-targeting CAR-T cell killing activity: MHCC97H cells have strong MET activation driven by MET^amp^; JHH5 cells are known to have HGF autocrine-mediated MET activation; C3A cells have a MET expression level comparable to JHH5 cells but without MET activation; SNU398 cells do not express MET (Fig. 2A). To test MET-dependent killing, HCC cells were co-cultured with NT or MET-CAR-T cells (n = 5, from HS) at E:T ratios ranging from 1.25:1 to 10:1 for 24 h., followed by HCC cell survival analysis. We found that both MET-CAR.CD28ζ and MET-CAR.4-1BBζ T cells potently killed MHCC97H, C3A, JHH5 cells in a dose dependent manner, but had no effect on SNU398 cells, demonstrating a specific killing of MET^+^ HCC cells, independent of MET pathway activation (Fig. 2B).

### Confocal imaging of MET-CAR-T cell killing activity against MHCC97H^***mCherry***^ cells in vitro

To visualize the specific attacking activity of MET-CAR-T cells against MET-positive tumor cells, MHCC97H^*mCherry*^ cells were co-cultured with MET-CAR-T cells prelabeled with MitoTracker green at an E:T = 10:1 (Fig. 2C) for confocal imaging. MET-CAR∆ T cells failed to kill MHCC97H cells which continued to migrate with time and resulted in increased cell number after 12 h. In contrast, the killing activity of MET-CAR.CD28ζ and MET-CAR.41BBζ T cells was evident within 6 h after co-culture when MHCC97H cells became round and detached from the culture dish. Significant cell death was observed at 12 h when MHCC97H cells were mostly aggregated with the MET-CAR.CD28ζ and MET-CAR.41BBζ T cells (Fig. 2C). We imaged killing activity of MET-CAR.4-1BBζ T cells at a lower magnification to display more of the MHCC97H cells (Fig. 2C, lower panel). Thus, both MET-CAR.CD28ζ and MET-CAR.4-1BBζ T cells can specifically recognize and kill MET^+^ tumor cells.

### Killing activity of HCC patient-derived MET-CAR-T cells

**Fig. 3:**
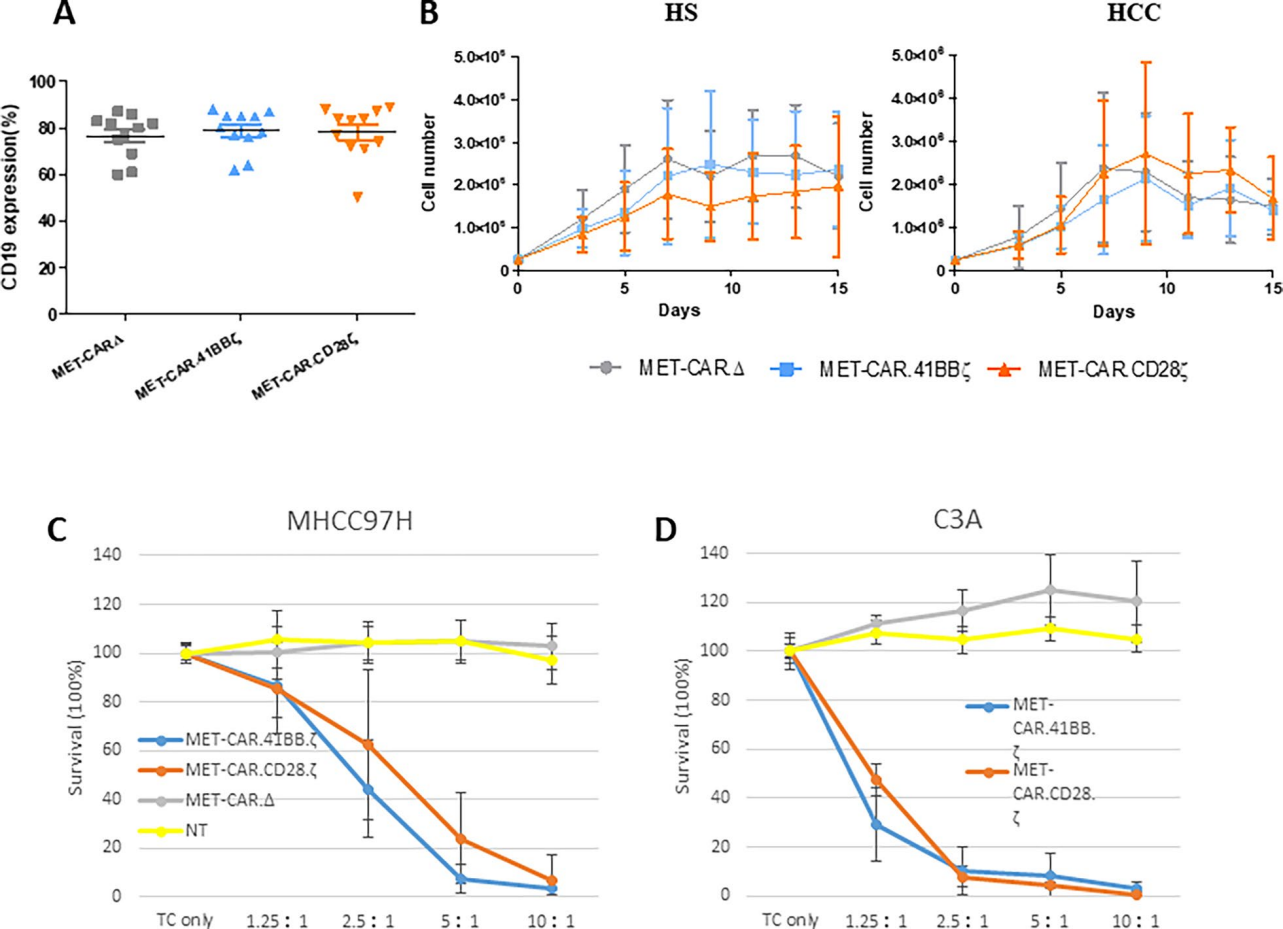
HCC patient-derived MET-CAR-T cell activity in vitro. **A** MET-CAR-T^HCC^ cell transduction efficacy determined by flow cytometry using CD19 as a tag. **B** In vitro growth curves of MET-CAR-T cells derived from HS- or HCC patient-derived PBMCs. Results were averaged from independent HS and HCC (n = 4) with duplicates. Short bar refers to standard deviation. **C**, **D** MET-CAR-T^HCC^ cell killing activity against MHCC97H (n = 8) and C3A (n = 2) in vitro. For MHCC97H cells (**C**), results were averaged from 8 independent HCC cases. For C3A cells (**D**), 2 independent HCC cases were averaged. Triplicates were used for each experiment. Bracketed bar refers to standard deviation

T cell dysfunction is common in advanced HCC [28–30]. Thus, we generated MET-CAR-T cells using PBMCs from HCC patients and tested their killing activity against MHCC97H and C3A cells. MET-CAR transduction efficacy (CD19%) in T cells from HCC patients ranged from 70 to 90% (Fig. 3A) and was comparable to the results from HS (Fig. 1C). MET-CAR-T cells from HS and HCC patients (n = 4) showed similar growth profiles (Fig. 3B). Comparing with HS results, HCC-derived MET-CAR.CD28ζ and MET-CAR.4-1BBζ T cells effectively killed MHCC97H cells at similar levels with E:T = 5:1 or higher (Fig. 3C, D). However, a reduced killing activity against MHCC97H cells was observed in MET-CAR-T cells derived from HCC patients with lower E:T ratio. We therefore tested killing activity against MHCC97H and C3A cells using HS and HCC-derived MET-CAR-T cells (n = 2) at low E:T ratio ranging from 0.625:1 to 5:1 (Additional file 1: Fig. S1). With MHCC97H cells, HS-derived MET-CAR.CD28ζ and MET-CAR.4-1BBζ T cells showed higher killing efficacy than those from HCC patients with E:T ratios = 2.5:1 and 1.25:1 (student *t* test, p < 0.05). When tested in C3A cells, the killing advantage of HS cases becomes small but is statistically significant when E:T = 2.5:1 (HS vs HCC, p < 0.05). Overall, HCC-derived MET-CAR-T cells demonstrate good killing activity against both MHCC97H and C3A cells.

### Multiple cytokines are secreted by MET-CAR-T cells upon activation

**Fig. 4:**
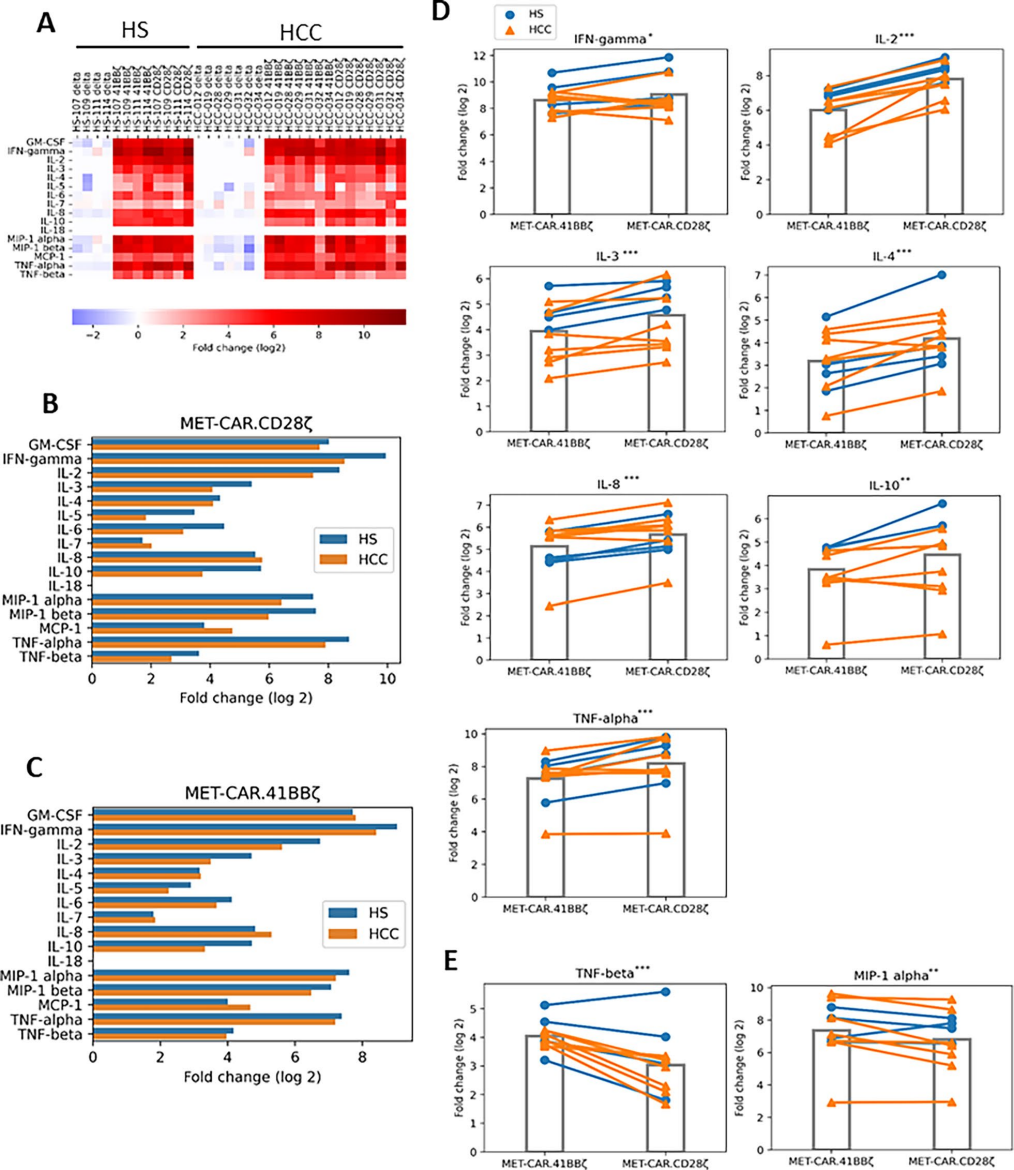
Cytokine release from MHCC97H-stimulated MET-CAR-T cells. **A** Heatmap analysis of multi-panel cytokine release by MET-CAR-T cells generated from HS and HCC patients. **B–C** Comparison of 16 cytokines released by MET-CAR.CD28ζ T cells (**B**) or MET-CAR.4-1BBζ T cells (**C**) between HS (n = 4) and HCC (n = 7) groups. **D–E** Cytokines that are produced significantly higher in MET-CAR.CD28ζ T cells (**D**) or MET-CAR.4-1BBζ T cells (**E**). *Paired t test, p < 0.01 only in HS group; **Paired t test, p < 0.05 in both HS and HCC groups; ***Paired t test, p < 0.01 in both HS and HCC groups

Upon antigen stimulation, activated CAR-T cells release cytokines to elicit their effector function such as cytolysis and/or promoting proliferation. To further evaluate MET-CAR-T cell performance, we co-cultured NT and 3 types of MET-CAR-T cells generated from HS (n = 4) and HCC (n = 7) patients with MHCC97H cells for 24 h and measured the production of 16 cytokines (Fig. 4, Additional file 2: Table S1). We observed significant up-regulation in the production of 15 out of 16 cytokines in both MET-CAR.CD28ζ, MET-CAR.4-1BBζ T cells when compared with MET-CARΔ T cells, demonstrating strong activation of both MET-CAR.CD28ζ and MET-CAR.4-1BBζ T cells (Fig. 4A). GM-CSF, IL-2, IFNγ, IL-6, TNFα and TNFβ are commonly expressed in activated T cells, indicating CAR-T cell activation and cytotoxicity. Overall, MET-CAR-T cells from HS showed a trend towards producing more of these cytokines than those from HCC patients (Fig. 4B, C, Additional file 2: Table S1), although additional samples are needed to conclusively validate this observation. CD28ζ and 4-1BBζ co-stimulatory signaling have different effector T cell functions, with CD28ζ exhibiting a more powerful cytotoxic effect while 4-1BBζ have longer persistence [31]. We therefore compared cytokine release between MET-CAR.CD28ζ and MET-CAR.4-1BBζ T cells (Fig. 4D, E). While MET-CAR.CD28ζ T cells produced significantly higher levels of 7 out of 16 cytokines, including IFNγ, IL-2, IL-3, IL-4, IL-8, IL-10, and TNFα (Fig. 4D), the MET-CAR.4-1BBζ T cells produced higher levels of TNFβ and MIP-1α (Fig. 4E). Notably, the 7 cytokines preferentially produced higher in MET-CAR.CD28ζ T cells are all typically associated with cytotoxicity, supporting an increased effector function of MET-CAR.CD28ζ in general.

### MET-CAR.CD28ζ is more potent than MET-CAR.4-1BBζ in anti-HCC tumor growth in vivo

**Fig. 5:**
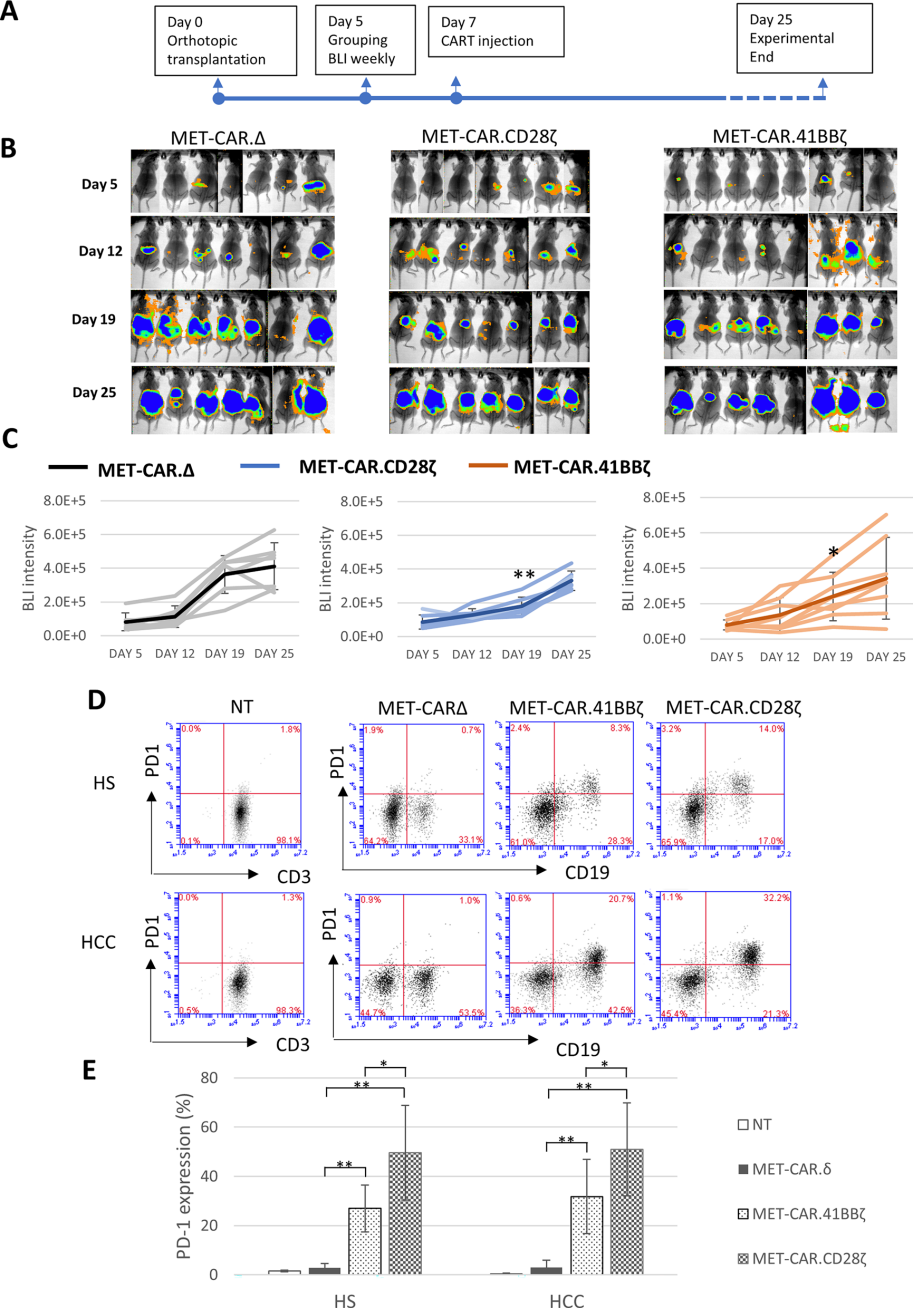
MET-CAR-T cell efficacy against MHCC97H orthotopic tumor growth *in nude mice*. **A** Time line of orthotopic tumor initiation and MET-CAR-T cell injection (*i.v*.). **B** MHCC97H orthotopic tumor growth measured by BLI. **C** Tumor growth curve plotted with BLI signal intensity of individual mice as shown in **B**. Darker line indicates the average signal intensity of each group. Bar with brackets refers to standard deviation. Note that at Day 19, BLI signal intensity in MET-CAR.CD28ζ and MET-CAR.4-1BBζ groups are significantly lower than that in MET-CAR∆ group (student *t* test, one tail, * p < 0.05; ** p < 0.01). **D** PD-1 expression level in NT and MET-CAR-T cells after 3-day co-culture with MHCC97H cells analyzed by flow cytometry. **E** Averaged results of PD-1 expression in MET-CAR-T cells from HS (n = 6) and HCC cases (n = 5). Short bar refers to standard deviation. (*paired *t* test, p < 0.01; **student *t* test, p < 0.01)

The human MHCC97H cell line has a higher copy number of MET^amp^ along with translocation from chromosome 7 to chromosomes 1 and 9, resulting in tumors with strong MET expression and ligand-independent MET activation, making it unique for studying MET-targeted therapy [20, 21]. To evaluate the therapeutic efficacy of MET-CAR-T cells in vivo, MHCC97H tumors expressing the luciferase reporter gene were implanted into nude mice orthotopically for treatment with MET-CAR-T cells (Fig. 5). Five days after transplantation, mice bearing MHCC97H^luc+^ tumors were randomized into 3 groups to receive a one-time, *i.v.* treatment of MET-CARΔ, MET-CAR.CD28ζ, or MET-CAR.4-1BBζ T cells (5 × 10^6^). The orthotopic tumor growth was measured by BLI once a week (Fig. 5A, B). Both MET-CAR.CD28ζ (student t test, two-tail, p < 0.01) and MET-CAR.4-1BBζ (student t test, one-tail, p < 0.05) T cells significantly inhibited MHCC97H orthotopic tumor growth by day 19, with MET-CAR.CD28ζ T cells exhibiting higher efficacy than MET-CAR.4-1BBζ T cells (Fig. 5C). This is consistent with the in vitro results showing that MET-CAR.CD28ζ T cells produce higher levels of cytokines than MET-CAR.4-1BBζ T cells in response to MHCC97H cell stimulation (Fig. 4).

Both MET-CAR.CD28ζ T cells and MET-CAR.4-1BBζ T cells showed potent killing activity against MHCC97H cells in vitro, but less effective anti-tumor efficacy in vivo, suggesting a suppressive regulation by tumor microenvironment. In advanced HCC, tumor cells express PD-1 ligand (PD-L1) which interacts with PD-1 expressed on effector T cells, leading to T cell exhaustion and dysfunction [30, 32]. To test whether MHCC97H cells may negatively regulate MET-CAR-T cell activity through PD-L1/PD-1 interaction, we co-cultured MHCC97H cells with NT and MET-CAR-T cells generated from HS (n = 6) or HCC patients (n = 5) for 3 days, then analyzed PD-1 expression in MET-CAR-T cells using flow cytometry. Co-culture with MHCC97H cells significantly increased PD-1 expression levels in MET-CAR.CD28ζ and MET-CAR.4-1BBζ T cells compared with NT and MET-CARΔ T cells (student t test, p < 0.001, Fig. 5, D, E). Moreover, MET-CAR.CD28ζ T cells demonstrated significantly higher PD-1 expression than MET-CAR.4-1BBζ T cells (HS: 49.6 ± 19.1 vs. 27.0 ± 16.5, HCC: 50.9 ± 18.9 vs. 31.8 ± 15.1, paired *t* test, p < 0.01) (Fig. 5E). These results suggest that although the CD28 costimulatory domain functions more potently than 4-1BB in activating MET-CAR-T cells, it also acts more in exacerbating MET-CAR-T cell exhaustion, resulting in reduced expansion, persistence and anti-tumor activity. Most patients enrolled in the study are scored as Child–Pugh Class A or B with liver functions suitable for operation, which maybe the reason that PD-1 upregulation in MET-CAR T cells from HCC donors are not significantly higher than that from HS donors.

### MET-CAR.CD28ζ T cell expansion, persistence and anti-tumor efficacy in vivo

**Fig. 6:**
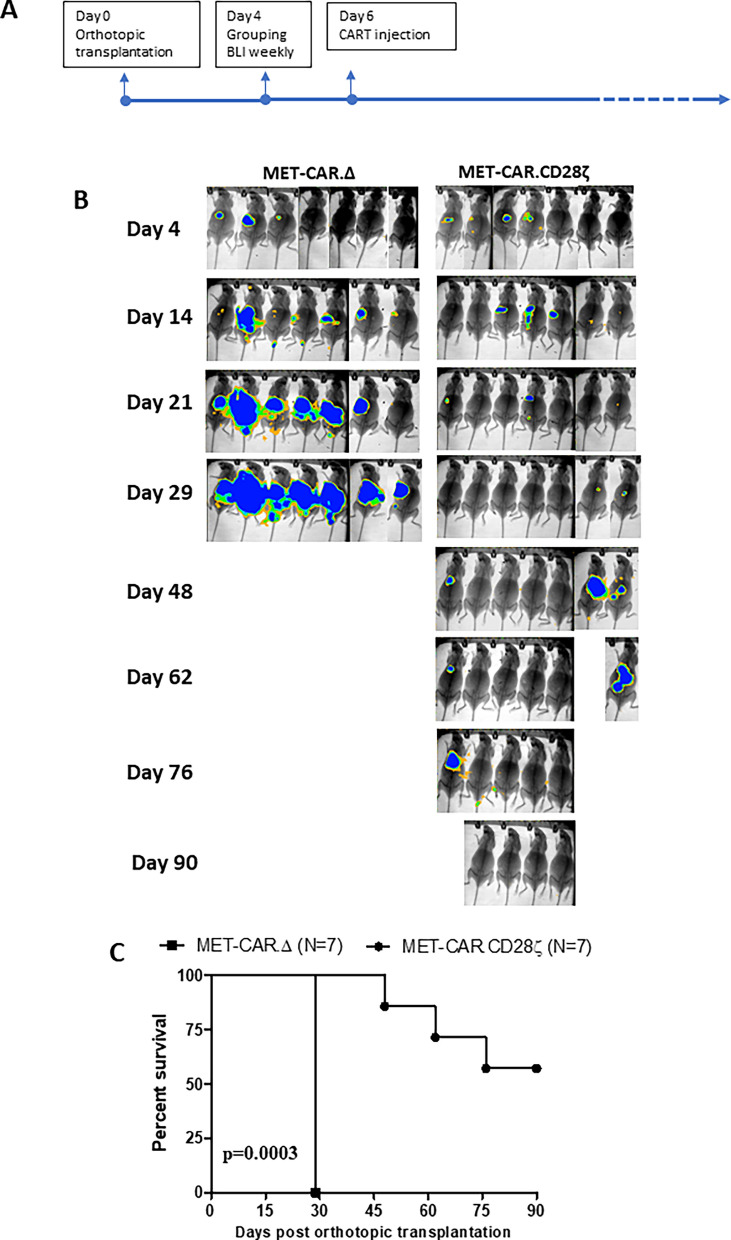
MET-CAR.CD28ζ T cells effectively inhibited MHCC97H tumor growth in NOD/SCID gamma mice. **A** Time line of orthotopic tumor initiation and MET-CAR-T cell injection (*i.v*.). **B** MHCC97H orthotopic tumor growth measured by BLI. **C** Survival time of MHCC97H orthotopic tumors treated by MET-CAR-T cells. (n = 7, *** p < 0.001)

Compared with 4-1BBζ, MET-CAR.CD28ζ T cells showed higher activity in vitro, and were more potent in vivo, and therefore were selected for further evaluation. NOD/SCID gamma mice were used for testing the therapeutic potential of MET-CAR.CD28ζ T cell activity against MHCC97H tumors, using MET-CAR∆ for control (Fig. 6A). A one-time tail-vein injection of MET-CAR.CD28ζ T cells (5 × 10^6^ cells) induced tumor regression after day 14 and significantly inhibited MHCC97H orthotopic tumor growth in 4 out of 7 mice for 90 days while control mice were sacrificed on day 29 due to tumor progression (Fig. 6B, C).

**Fig. 7:**
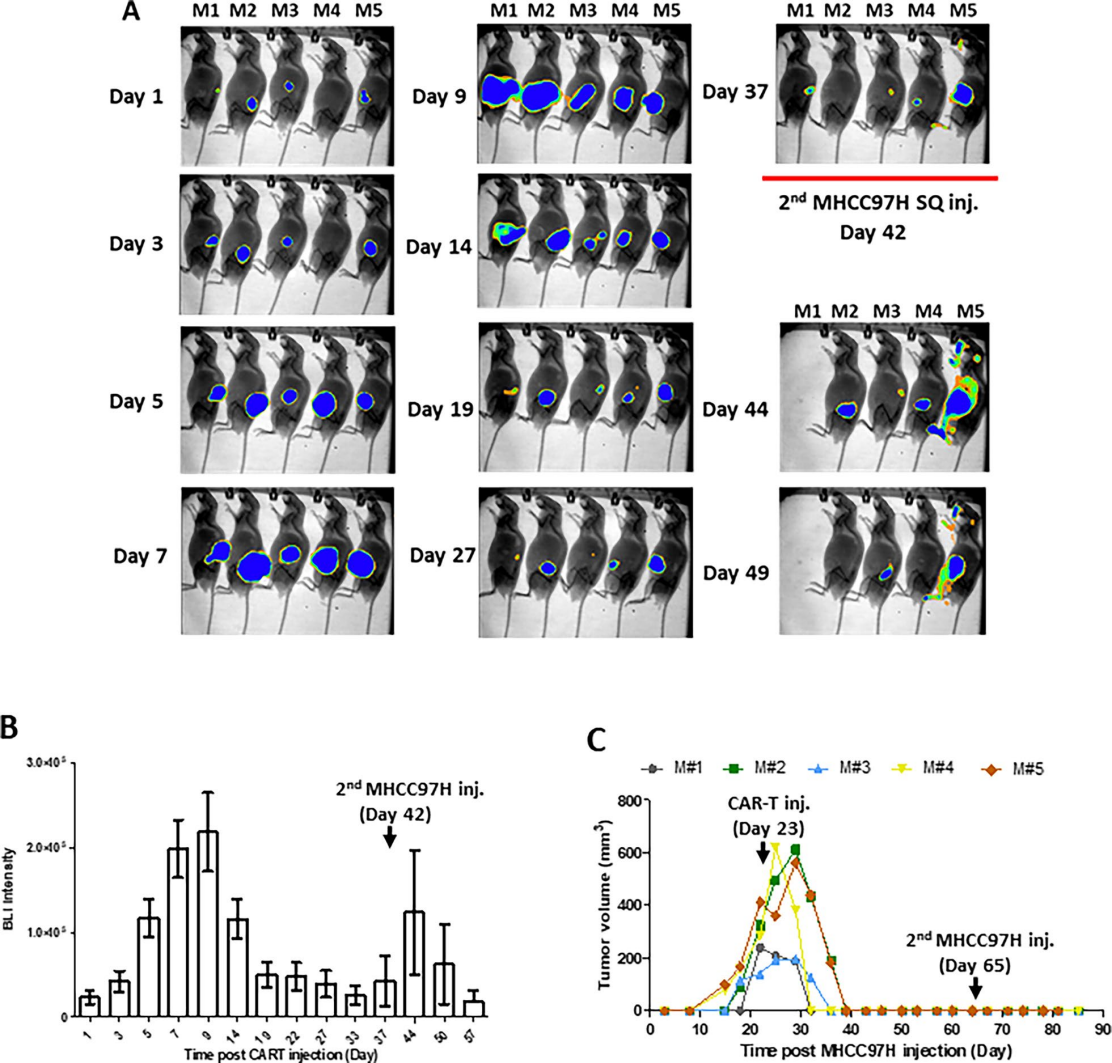
Real-time imaging of MET-CAR.CD28ζ-Luc T cell expansion and persistence in vivo (n=5). **A** Images of MHCC97H SQ mice after CAR-T cell injection. **B** BLI signal intensity analyzed from A. **C** Tumor volume of individual mouse from A as measured by caliper. V = length x width x depth (mm^3^).  Note that CAR-T cells were injected 23 days after MHCC97H injection

To evaluate MET-CAR-T cell expansion and persistence in vivo, MHCC97H cells were SQ injected into mice to initiate tumor growth followed by intra-tumoral injection of MET-CAR.CD28ζ T cells co-transduced with a GFP-Luc plasmid to real-time monitor CAR-T cells using BLI. MET-CAR∆ was used as control (Additional file 3: Fig S2). We found that one-time injection of MET-CAR.CD28ζ T cells completely inhibited tumor growth as compared with MET-CAR∆ T cells (Additional file 3: Fig S2A). Correspondingly, BLI signaling intensity rapidly increased in mice injected with MET-CAR.CD28ζ T cells on day 4, reached peak on day 6 and decreased thereafter as tumors regressed, indicating an effective T cell expansion upon antigen stimulation. In contrast, the BLI signal intensity in MET-CAR∆ group of mice did not increase until later days possibly due to a non-specific expansion in response to tumor growth (Additional file 3: Fig S2B, C). To better profile MET-CAR.CD28ζ T cells in vivo*,* another set of 5 mice bearing MHCC97H SQ tumors were used for the same analysis with PBMCs from a different donor (Fig. 7). We observed a significant increase of BLI signal intensity within the first 10 days after intra-tumoral injection of MET-CAR.CD28ζ T cells (Fig. 7A, B). In parallel, tumor regression started 7 days after MET-CAR-T cell injection (Fig. 7C). Again these data demonstrate a potent activation, expansion, and killing activity of MET-CAR.CD28ζ T^GFP−Luc^ cells upon antigen stimulation in vivo. MET-CAR.CD28ζ T^GFP−Luc^ cell expansion started to decrease 14 days after injection due to the lack of antigen stimulation resulted from tumor regression. However, a low level of sustained BLI signal intensity was enriched in the local area of injection even after the complete regression of MHCC97H tumors, suggesting a formation of memory T cells (Fig. 7A, B). To test whether the residual MET-CAR.CD28ζ T^GFP−Luc^ cells have memory response and remain anti-tumor activity, a re-challenge experiment was performed by injecting MHCC97H cells again on both sides of the animals at day 42 (Fig. 7A). We observed a transient MET-CAR.CD28ζ T^GFP−Luc^ cell expansion as indicated by the increase of BLI signal intensity (Fig. 7A, B). Importantly, tumor formation was not observed during the additional 30 days until the experiment was terminated (Fig. 7C).

## Discussion

MET is a promising anti-HCC target; however, the efficacy of MET inhibitors in clinical trials remains controversial. Results from preclinical animal models or human clinical trials suggest that effective MET inhibitors require tumors to be driven by an active form of MET (phosphor-MET), [19, 20, 25, 33–36]. In contrast, there are solid tumors with high total MET expression instead of phosphor-MET that fail to respond to MET inhibitors [19, 20]. As such, specific MET kinase inhibitors still have limited application. Furthermore, because many of the MET downstream pathways also are controlled by other RTKs, long-term treatment with specific MET inhibitors may cause cross activation of other RTK signaling, a common feature of drug resistance [15, 37–39]. Compared with MET inhibitors, MET-targeting CAR-T cells have the advantage of targeting tumors based on total MET expression regardless of downstream pathway activities. Here, we show that MetMab-based MET-CAR.CD28ζ and MET-CAR.4-1BBζ T cells not only effectively killed MHCC97H (MET^amp^) cells, but also killed JHH5 cells that are driven by HGF-autocrine activation that are sensitive to MET inhibitors [21] and C3A cells overexpressing total MET (similar level to JHH5) but without MET activation, which showed no response to MET inhibitors previously [20]. In contrast, SNU398 cells without MET expression showed no response to MET inhibitors nor to either MET-CAR.CD28ζ or MET-CAR.4-1BBζ T cells (Fig. 2A, B). Compared with MET TKIs and neutralizing antibodies, MET-CAR-T cells may selectively target MET^+^ tumors with total MET expression as a biomarker. Because MET-CAR-T cells function through T cell-mediated cytotoxicity rather than MET RTK signaling, the efficacy of MET-CAR-T cells is independent of MET pathway activation.

While CD8^+^ cytotoxic T cells are the paradigm phenotype of T cell cytotoxicity, our MET-CAR.CD28ζ and MET-CAR.4-1BBζ T cells are a mixture of CD4^+^ and CD8^+^ T cells, with CD4^+^ CAR-T cells constituting 60–70% of the population in MET-CAR-T cells (Fig. 1B). Both MET-CAR.CD28ζ and MET-CAR.4-1BBζ T cells effectively killed HCC cells at an E:T = 2:1, suggesting that CD4^+^ MET-CAR-T cells are functional with cytotoxic activity. A recent study with Her2-targeting CAR-T cells reported that after OKT3/CD28 stimulation, the transduced CAR-T cells consistently included a mixed population of CD4^+^ T cells and CD8^+^ T cells, of which CD4^+^ T cells were the major subset. Both subsets of Her2-CAR-T cells demonstrated strong cytotoxic activity, with CD4^+^ T cells showing higher longevity than CD8^+^ T cells [26]. A glioblastoma study using CAR-T cell therapy targeting IL-13 receptor α2 (IL13Rα2) found that with IL13Rα2^+^ stimulation the CD8^+^ CAR-T cells demonstrated robust but short-term effector function and became exhausted more quickly, while CD4^+^ CAR-T cells outperformed CD8^+^ CAR-T cells with a superior antitumor response. This is consistent in CAR-T cells derived from both HS and GBM patients [27]. Taken together, our results suggest that both CD4^+^ and CD8^+^ subtypes of MET-CAR-T cells can be responsible for the anti-tumor efficacy, and that CD4^+^ MET-CAR-T cells may elicit more robust effector T cell functions when applied to clinical settings.

Among the several co-stimulatory domains, CD28 and 4-1BB are the most common ones used for designing CARs. Although their biological functions (T cell proliferation, cytokine production and tumor cell killing) are similar, CD28 and 4-1BB domains differ in the speed and the level of downstream signaling pathway activation and amplification upon antigen stimulation [31, 40]. While CARs containing the CD28 domain generate a faster, stronger signal than those containing the 4-1BB domain, leading to a more potent target cell killing activity, CD28-CARs also demonstrated limited long-term survival and anti-tumor durability due to CAR-T cell exhaustion [41, 42]. In our study, MET-CAR.CD28ζ T cells demonstrated significantly higher PD-1 expression than MET-CAR.4-1BBζ T cells after tumor cell stimulation (Fig. 5D, E), suggesting a “faster response, faster exhaustion” phenotype. These may explain the results from our study that upon MET-positive tumor cell stimulation, MET-CAR.CD28ζ T cells overall produced higher levels of cytokines than MET-CAR.4-1BBζ T cells in vitro, significantly inhibited MHCC97H tumor growth with higher efficacy than MET-CAR.4-1BBζ T cells in vivo for the first 2 weeks, but the difference of growth inhibition diminished thereafter (Fig. 5B, C). While the use of NOD/SCID gamma mice significantly improved expansion, persistence, and therapeutic efficacy of MET-CAR.CD28ζ T cells in vivo, either due to a better host immune-deficient environment or a better donor of PBMCs, resistance to treatment was observed at a later stage (Fig. 6). So far, the best efficacy observed was based on SQ tumor model, by which one-time intra-tumoral injection of MET-CAR.CD28ζ T cells induced tumor regression without showing recurrence (Fig. 7), which is more potent than other reported MET-CARs requiring multiple injections at tumor site to be effective [43, 44]. While this delivery approach eliminated the challenge for T cells to traffic and penetrate into the tumors, in the context of HCC, it is feasible to perform ultrasound-guided local delivery of CAR-T cells. Taken together, our results suggest that while MET-CAR.CD28ζ is more potent than MET-CAR.4-1BBζ in anti-HCC activity for further development, approaches to overcome MET-CAR.CD28ζ T cell exhaustion need to be considered to improve its long-term efficacy.

Among the several CAR-T cell targets developed for HCC, glypican-3 (GPC3) targeting CAR-T cell therapy has entered clinical trials of HCC patients showing promising results [45, 46]. GPC3 is a plasma membrane-bound proteoglycan specially overexpressed in HCC but not in normal liver tissues, making it an ideal target for CAR-T cell therapy in HCC [47]. Although GPC3-CAR-T cells showed anti-tumor activity, the effect was reduced significantly in the presence of GPC3 shed off the tumor cell surfaces [48]. Approximately 40% of HCC patients exhibited significant amounts of shed GPC3 in their serum [47]. Since circulating MET ectodomain shedding is also reported in preclinical cancer cell models and lung cancer patients [49, 50], how soluble MET shedding would influence MET-CAR-T cell efficacy needs to be evaluated when MET-CAR-T cells enter clinical trials.

Unlike hematological malignancy, CAR-T cell therapy has not yet been successful in solid tumors. While immune suppressive tumor microenvironment produces inhibitory cytokines, regulatory modulators and co-inhibitory receptors that challenge immune cell responsiveness, the low tumor infiltration of T cells can further challenge the anti-tumor efficacy in vivo [18, 51]. Thus, efforts to block negative signaling and revert T cell exhaustion should improve CAR-T cell efficacy. To date, two PD-L1/PD-1 inhibitors (nivolumab and pembrolizumab) are FDA approved for treating advanced HCC [12]. Another checkpoint inhibitor (tremelimumab) developed for targeting cytotoxic T-lymphocyte associated protein 4 (CTLA-4) also showed promising clinical results for treating HCC patients [52]. Of note, genetic editing of CAR vectors to transcriptionally suppress PD-1 expression or adding PD-1 blockage as a combination strategy was able to improve CAR-T cell persistence and anti-tumor efficacy in vivo [26, 53]. Thus, future optimization to overcome MET-CAR-T cell exhaustion via PD-L1/PD-1 and other essential inhibitory signaling pathways such as CTLA-4 [52] and LAG3 [54] could improve MET-CAR-T cell therapeutic efficacy.

In summary, we generated and characterized MetMab-based MET-specific CAR-T cells for the therapeutic potential for targeting MET^+^ HCC. Compared with MET-TKIs and neutralizing antibodies targeting MET activation, MET-CAR-T cells recognize and kill HCC cells based on total MET expression, and the activity is independent of MET signaling pathway activity. MET-CAR.CD28ζ demonstrated a higher level of activating effector T cell functions and anti-tumor efficacy than MET-CAR.4-1BBζ. While MET-CAR.CD28ζ is preferred for future development, the architecture of the CAR construct as well as strategies to compensate for tumor microenvironment-mediated CAR-T cell exhaustion are necessary to enhance MET-CAR-T cell therapeutic efficacy.

### Supplementary Information

**Additional file 1: Figure S1.** Killing activity comparison of MET-CAR-T cells derived from HS and HCC donors. **A**–**B** Killing activity of MET-CAR-T cells derived from HS (**A**) and HCC (**B**) donors against MHCC97H cells (n=2). **C**–**D** Killing activity of MET-CAR-T cells derived from HS (**C**) and HCC (**D**) donors against C3A (n=2). (HS vs. HCC, student t test, *p<0.01; **p<0.001).

**Additional file 2: Table S1.** Multi-panel cytokine released by MET-CAR T cells after coculture with MHCC97H cells.

**Additional file 3: Figure S2.** Real-time imaging of MET-CAR T cell expansion and persistence in vivo. **A** Average tumor volume of 5 mice from C as measured by caliper. V=length x width x depth (mm^3^). **B** Normalized BLI intensity analyzed From C. **C** Images of MHCC97H SQ mice after CAR T cell injection. One mouse died because of anesthesia on day 10.

## Data Availability

Data are available upon reasonable request.

## Abbreviations

BLI: Bioluminescence imaging
CAR: Chimeric antigen receptor
CTLA-4: Cytotoxic T-lymphocyte associated protein 4
DIC: Differential interference contrast
E:T: Effector T cell: Tumor cell
GPC3: Glypican-3
HBV/HCV: Hepatitis B or C virus
HCC: Hepatocellular carcinoma
HGF: Hepatocyte growth factor
HS: Healthy subjects
IL13Rα2: IL-13 receptor α2
*i.v*: Intravenous
MAP: Multi-Analyte Profile A
MET^*amp*^: MET amplification
MetMab: Anti-MET monoclonal antibody
NT: Non-transduced
PBMCs: Peripheral blood mononuclear cells
PD-1: Programmed cell death protein 1
PD-L1: PD-1 ligand
RTK: Receptor tyrosine kinases
SSR: Short non-signaling spacer domain region
SQ: Subcutaneously
TCR: T cell receptor
TKI: Tyrosine kinase inhibitor
TM: TCR transmembrane
