# Supplementary material for: Tyrosine kinase signaling-independent MET-targeting with CAR-T cells
Source: J Transl Med. 2023 Oct 1;21:682. doi: 10.1186/s12967-023-04521-9 (PMC10544186; doi:10.1186/s12967-023-04521-9)
Supplement: Supplementary file 1 — Additional file 1: Figure S1. Killing activity comparison of MET-CAR-T cells derived from HS and HCC donors. A–B Killing activity of MET-CAR-T cells derived from HS (A) and HCC (B) donors against MHCC97H cells (n=2). C–D Killing activity of MET-CAR-T cells derived from HS (C) and HCC (D) donors against C3A (n=2). (HS vs. HCC, student t test, *p<0.01; **p<0.001). [file 12967_2023_4521_MOESM1_ESM.docx]

**
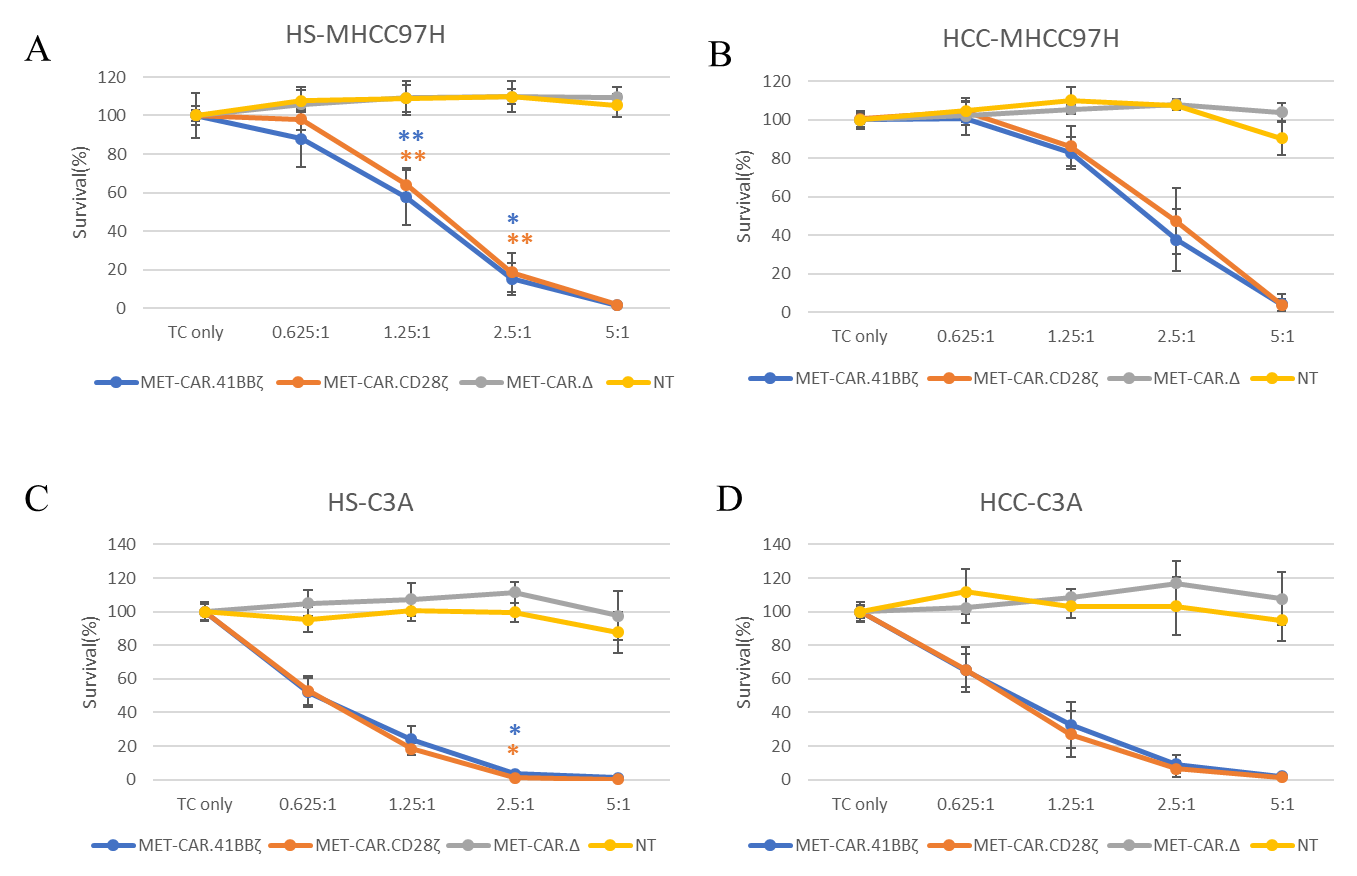
**

**Fig.S1 Killing activity comparison of MET-CAR-T cells derived from HS and HCC donors. (**A-B) Killing activity of MET-CAR-T cells derived from HS (A) and HCC (B) donors against MHCC97H cells (n=2). (C-D) Killing activity of MET-CAR-T cells derived from HS (C) and HCC (D) donors against C3A (n=2). (HS vs. HCC, student t test, *p<0.01; **p<0.001)
