# Supplementary material for: Tyrosine kinase signaling-independent MET-targeting with CAR-T cells
Source: J Transl Med. 2023 Oct 1;21:682. doi: 10.1186/s12967-023-04521-9 (PMC10544186; doi:10.1186/s12967-023-04521-9)
Supplement: Supplementary file 2 — Additional file 2: Table S1. Multi-panel cytokine released by MET-CAR T cells after coculture with MHCC97H cells. [file 12967_2023_4521_MOESM2_ESM.docx]

**Table S1** Multi-panel cytokine released by MET-CAR T cells after coculture with MHCC97H cells.

|  |  | HS (n=4) | | HCC (n=7) | |
| --- | --- | --- | --- | --- | --- |
| GM-CSF | NT | 13.75 ± | 5.68 | 9.11± | 5.69 |
|  | MET-CARΔ | 9.15 ± | 2.98 | 6.80± | 0.00 |
|  | MET-CAR.41BBζ | 2980.00 ± | 1243.95 | 2020.14± | 751.45 |
|  | MET-CAR.CD28ζ | 3867.50 | 2251.41 | 2135.71± | 1531.11 |
| IFN-γ | NT | 1.25 ± | 0.24 | 1.14± | 0.11 |
|  | MET-CARΔ | 1.38 ± | 0.38 | 1.49± | 1.02 |
|  | MET-CAR.41BBζ | 877.00 ± | 770.79 | 428.29± | 191.21 |
|  | MET-CAR.CD28ζ | 1860.00 ± | 1851.25 | 564.14± | 583.11 |
| IL-2 | NT | 15.00 ± | 0.00 | 15.00± | 0.00 |
|  | MET-CARΔ | 15.00 ± | 0.00 | 15.00± | 0.00 |
|  | MET-CAR.41BBζ | 1678.00 ± | 518.92 | 1009.71± | 792.38 |
|  | MET-CAR.CD28ζ | 5307.50 ± | 2112.41 | 3221.57± | 2036.55 |
| IL-3 | NT | 1.20 ± | 0.00 | 1.20± | 0.00 |
|  | MET-CARΔ | 1.20 ± | 0.00 | 1.20± | 0.00 |
|  | MET-CAR.41BBζ | 34.75 ± | 19.4 | 17.43± | 13.51 |
|  | MET-CAR.CD28ζ | 53.00 ± | 17.07 | 28.56± | 28.19 |
| IL-4 | NT | 11.25 ± | 12.50 | 5.00± | 0.00 |
|  | MET-CARΔ | 5.50 ± | 1.00 | 5.00± | 0.00 |
|  | MET-CAR.41BBζ | 117.75 ± | 111.32 | 62.34± | 42.23 |
|  | MET-CAR.CD28ζ | 293.75 ± | 297.26 | 105.00± | 60.80 |
| IL-5 | NT | 21.90 ± | 39.40 | 3.36± | 2.77 |
|  | MET-CARΔ | 7.40 ± | 10.40 | 2.30± | 0.26 |
|  | MET-CAR.41BBζ | 97.93 ± | 109.93 | 21.57± | 18.80 |
|  | MET-CAR.CD28ζ | 291.78 ± | 390.25 | 14.21± | 9.58 |
| IL-6 | NT | 1.25 ± | 0.86 | 0.85± | 0.73 |
|  | MET-CARΔ | 1.24 ± | 0.93 | 1.10± | 1.50 |
|  | MET-CAR.41BBζ | 29.03 ± | 29.39 | 10.54± | 6.47 |
|  | MET-CAR.CD28ζ | 37.15 ± | 33.91 | 11.17± | 14.58 |
| IL-7 | NT | 7.50 ± | 0.00 | 8.00± | 1.32 |
|  | MET-CARΔ | 8.40 ± | 0.60 | 10.86± | 4.79 |
|  | MET-CAR.41BBζ | 28.00 ± | 13.22 | 31.71± | 15.74 |
|  | MET-CAR.CD28ζ | 28.00 ± | 14.72 | 58.50± | 84.87 |
| IL-8 | NT | 60.75 ± | 24.02 | 79.71± | 126.50 |
|  | MET-CARΔ | 46.50 ± | 20.14 | 70.71± | 112.70 |
|  | MET-CAR.41BBζ | 1672.50 ± | 444.33 | 1805.71± | 438.67 |
|  | MET-CAR.CD28ζ | 2760.00 ± | 826.60 | 2610.00± | 1091.91 |
| IL-10 | NT | 2.10 ± | 0.00 | 2.10± | 0.00 |
|  | MET-CARΔ | 2.10 ± | 0.00 | 2.10± | 0.00 |
|  | MET-CAR.41BBζ | 55.50 ± | 2.38 | 27.03± | 16.40 |
|  | MET-CAR.CD28ζ | 122.50 ± | 63.45 | 41.63± | 34.35 |
| IL-18 | NT | 11.00 ± | 0.00 | 11.00± | 0.00 |
|  | MET-CARΔ | 11.00 ± | 0.00 | 11.00± | 0.00 |
|  | MET-CAR.41BBζ | 11.00 ± | 0.00 | 11.00± | 0.00 |
|  | MET-CAR.CD28ζ | 11.00 ± | 0.00 | 11.14± | 0.38 |
| MIP-1 α | NT | 36.00 ± | 31.19 | 53.57± | 48.09 |
|  | MET-CARΔ | 32.50 ± | 30.78 | 33.86± | 19.04 |
|  | MET-CAR.41BBζ | 5762.50 ± | 2209.29 | 6161.43± | 2514.98 |
|  | MET-CAR.CD28ζ | 5467.50 ± | 2438.20 | 3518.57± | 1870.76 |
| MIP-1 β | NT | 273.00 ± | 285.45 | 387.14± | 417.17 |
|  | MET-CARΔ | 205.75 ± | 229.34 | 165.57± | 89.66 |
|  | MET-CAR.41BBζ | 28600.00 ± | 11987.49 | 27337.14± | 11023.69 |
|  | MET-CAR.CD28ζ | 49550.00 ± | 43186.15 | 19517.14± | 11877.35 |
| MCP-1 | NT | 28.25 ± | 26.50 | 46.14± | 39.40 |
|  | MET-CARΔ | 26.75 ± | 23.50 | 34.86± | 16.44 |
|  | MET-CAR.41BBζ | 570.75 ± | 686.14 | 1088.86± | 442.51 |
|  | MET-CAR.CD28ζ | 600.00 ± | 866.69 | 1153.86± | 468.98 |
| TNF-α | NT | 7.28 ± | 2.30 | 7.64± | 6.27 |
|  | MET-CARΔ | 5.68 ± | 1.86 | 4.91± | 1.76 |
|  | MET-CAR.41BBζ | 1393.25 ± | 853.75 | 1022.71± | 469.91 |
|  | MET-CAR.CD28ζ | 3636.25 ± | 2519.12 | 1988.86± | 1433.43 |
| TNF-β | NT | 6.30 ± | 0.00 | 6.30± | 0.00 |
|  | MET-CARΔ | 6.30 ± | 0.00 | 6.30± | 0.00 |
|  | MET-CAR.41BBζ | 129.00 ± | 70.32 | 99.57± | 15.79 |
|  | MET-CAR.CD28ζ | 120.25 ± | 126.85 | 44.14± | 17.85 |

MHCC97H cells were seeded in 24 well plates (10^5^ cells/well) for co-culture with NT or MET-CAR-T cells at 2:1 E:T ratio. After 24 hrs, conditioned medium from each well was collected, frozen and tested for a panel of 16 cytokines (pg/ml) using Human Cytokine Map A platform at Myriad RMB. Each data represents for Mean ± STDEV from healthy subjects (n=4) and HCC patients (n=7). Heat-map and statistics analysis is described in Fig 4.
