# Supplementary material for: Tyrosine kinase signaling-independent MET-targeting with CAR-T cells
Source: J Transl Med. 2023 Oct 1;21:682. doi: 10.1186/s12967-023-04521-9 (PMC10544186; doi:10.1186/s12967-023-04521-9)
Supplement: Supplementary file 3 — Additional file 3: Figure S2. Real-time imaging of MET-CAR T cell expansion and persistence in vivo. A Average tumor volume of 5 mice from C as measured by caliper. V=length x width x depth (mm3). B Normalized BLI intensity analyzed From C. C Images of MHCC97H SQ mice after CAR T cell injection. One mouse died because of anesthesia on day 10. [file 12967_2023_4521_MOESM3_ESM.docx]

**B**

**A**

**C**

MET-CAR.CD28ζ

MET-CAR.Δ


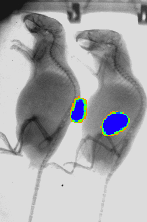

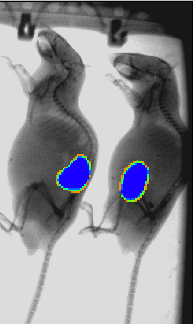

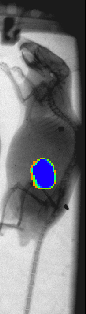

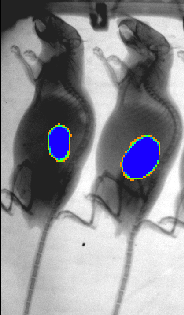

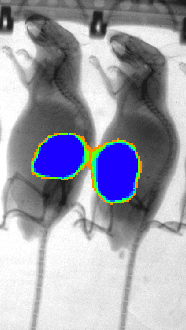

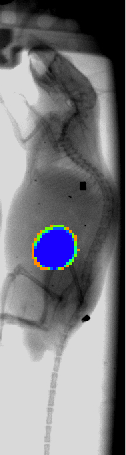

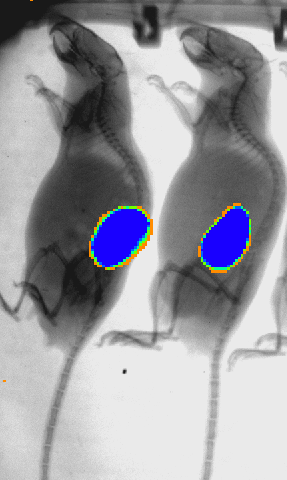

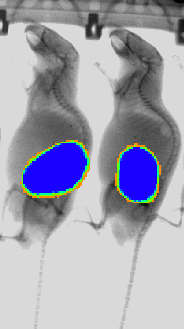

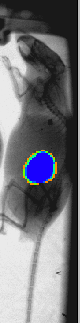

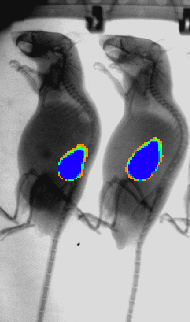

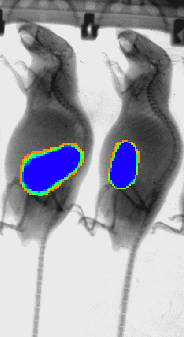

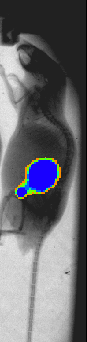

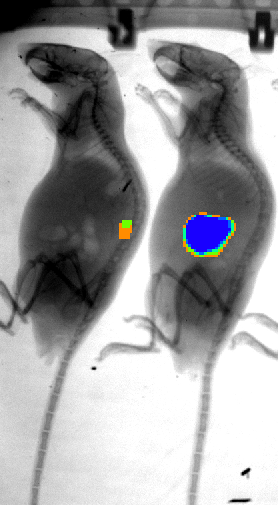

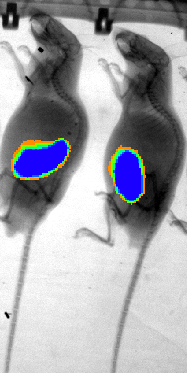

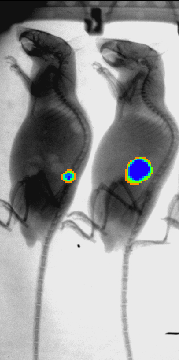

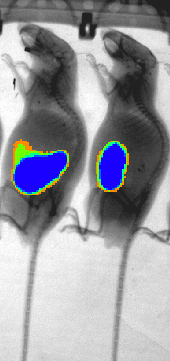

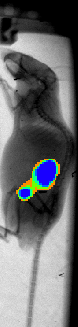

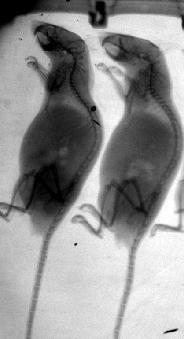

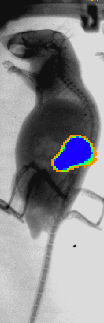

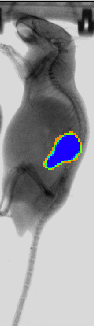

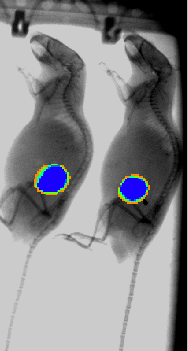

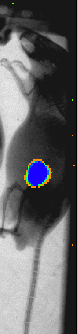

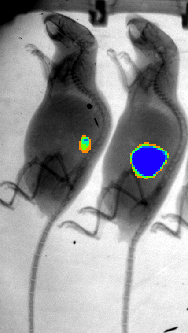

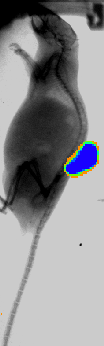

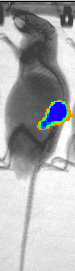

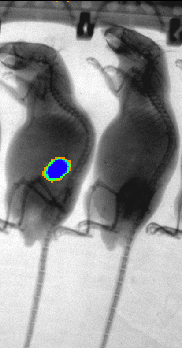

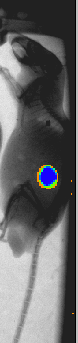

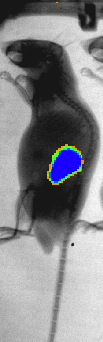

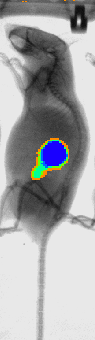

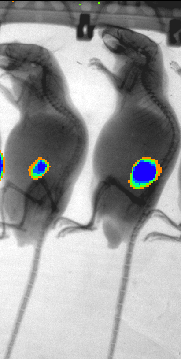

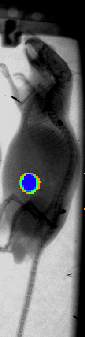

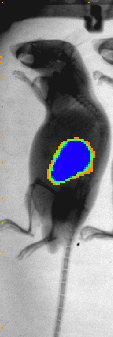

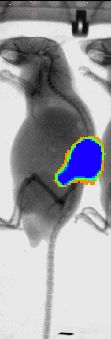

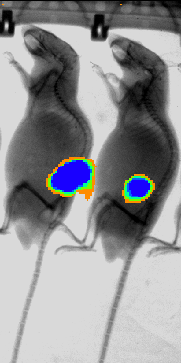

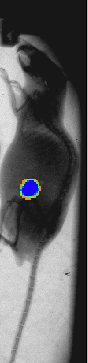

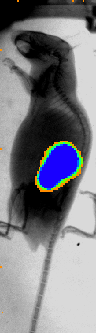

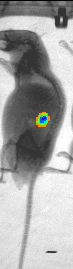

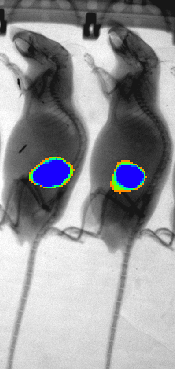

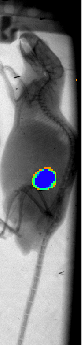

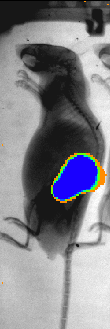

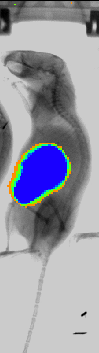

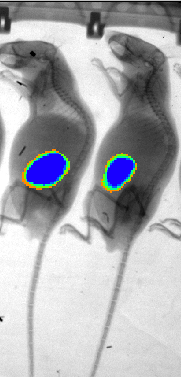

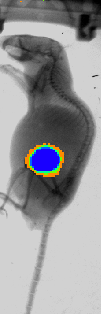

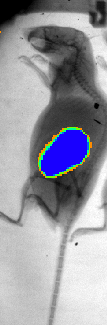

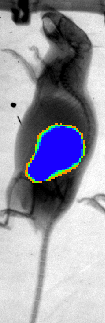

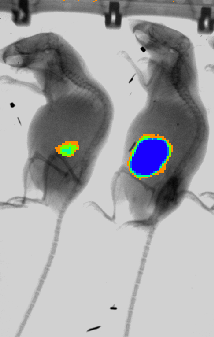

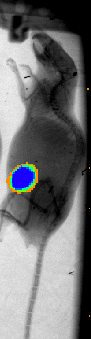


**Day 2**

**Day 4**

**Day 6**

**Day 8**

**Day 10**

**Day 12**

**Day 14**

**Fig.S2 Real-time imaging of MET-CAR T cell expansion and persistence in vivo. (**A) Average tumor volume of 5 mice from C as measured by caliper (V=length x width x depth (mm^3^). (B) Normalized BLI intensity analyzed From C. (C) Images of MHCC97H SQ mice after CAR T cell injection. One mouse died because of anesthesia on day 10.
